# Supplementary material for: A comprehensive experimental comparison between federated and centralized learning
Source: Database (Oxford). 2025 Mar 19;2025:baaf016. doi: 10.1093/database/baaf016 (PMC11928227; doi:10.1093/database/baaf016)
Supplement: baaf016_Supp [file baaf016_supp.zip › suppl_data/Suppl_Table_1.docx]

|  | Layer 1 | Layer 2 | Layer 3 |
| --- | --- | --- | --- |
| MNIST2 | Fully Connected: 784 x 100 | ReLU | Fully Connected: 100 x 2 |
| MNIST4 | Fully Connected: 784 x 100 | ReLU | Fully Connected: 100 x 4 |
| fashion MNIST | Fully Connected: 784 x 100 | ReLU | Fully Connected: 100x10 |
| AML | Fully Connected:  100 x 100 | ReLU | Fully Connected: 100x2 |
