# Supplementary material for: A comprehensive experimental comparison between federated and centralized learning
Source: Database (Oxford). 2025 Mar 19;2025:baaf016. doi: 10.1093/database/baaf016 (PMC11928227; doi:10.1093/database/baaf016)
Supplement: baaf016_Supp [file baaf016_supp.zip › suppl_data/Suppl_table_2.docx]

|  | Layer 1 | Layer 2 | Layer 3 |
| --- | --- | --- | --- |
| MNIST2 | Convolutional: kernel size:3 stride: 1 padding: 1 | Max pool: kernel = 2 stride = 2 | Fully Connected: 196 x 2 |
| MNIST4 | Convolutional: kernel size:3 stride: 1 padding: 1 | Max pool: kernel = 2 stride = 2 | Fully Connected: 196 x 4 |
| fashion MNIST | Convolutional: kernel size:3 stride: 1 padding: 1 | Max pool: kernel = 2 stride = 2 | Fully Connected: 196 x 10 |
| AML | Convolutional: kernel size:3 stride: 1 padding: 1 | Max pool: kernel = 2 stride = 2 | Fully Connected: 25 x 2 |
