# Supplementary material for: A comprehensive experimental comparison between federated and centralized learning
Source: Database (Oxford). 2025 Mar 19;2025:baaf016. doi: 10.1093/database/baaf016 (PMC11928227; doi:10.1093/database/baaf016)
Supplement: baaf016_Supp [file baaf016_supp.zip › suppl_data/Suppl_table_3.docx]

|  |  | KDR |  | ABL1 |  |
| --- | --- | --- | --- | --- | --- |
|  |  | Final accuracy (std) | Mean AUC (std) | Final accuracy (std) | Mean AUC (std) |
| LR | Central | 0.75 (0.007) | 0.69 (0.006) | 0.78 (0.01) | 0.75 (0.012) |
|  | Federated | 0.74 (0.004) | 0.68 (0.005) | 0.78 (0.01) | 0.74 (0.018) |
| SVM | Central | 0.76 (0.007) | 0.7 (0.004) | 0.78 (0.015) | 0.75 (0.017) |
|  | Federated | 0.74 (0.005) | 0.69 (0.007) | 0.77 (0.01) | 0.74 (0.017) |
| FNN | Central | 0.75 (0.002) | 0.68 (0.002) | 0.73 (0.004) | 0.71 (0.0) |
|  | Federated | 0.75 (0.005) | 0.68 (0.004) | 0.72 (0.003) | 0.71 (0.0) |
| CNN | Central | 0.72 (0.061) | 0.65 (0.053 | 0.76 (0.014) | 0.74 (0.015) |
|  | Federated | 0.78 (0.009) | 0.73 (0.012) | 0.77 (0.005) | 0.76 (0.001) |
| GBDT | Central | 0.82 (0.0) | 0.79 (0.0) | 0.81 (0.001) | 0.79 (0.001) |
|  | Federated | 0.73 (0.009) | 0.69 (0.003) | 0.78 (0.011) | 0.75 (0.005) |
