# Supplementary material for: A comprehensive experimental comparison between federated and centralized learning
Source: Database (Oxford). 2025 Mar 19;2025:baaf016. doi: 10.1093/database/baaf016 (PMC11928227; doi:10.1093/database/baaf016)
Supplement: baaf016_Supp [file baaf016_supp.zip › suppl_data/Suppl_table_4.docx]

|  |  |  | MNIST2 |  | MNIST4 |  |
| --- | --- | --- | --- | --- | --- | --- |
|  |  |  | Final accuracy (std) | Mean AUC (std) | Final accuracy (std) | Mean AUC (std) |
| LR | Central |  | 0.97 (0.001) | 0.97 (0.001) | 0.93 (0.001) | 0.91 (0.001) |
|  | Federated | IID | 0.97 (0.002) | 0.97 (0.002) | 0.93 (0.001) | 0.91 (0.001) |
|  |  | CI | 0.97 (0.001) | 0.96 (0.002) | 0.93 (0.001) | 0.91 (0.001) |
|  |  | SI | 0.97 (0.002) | 0.97 (0.002) | 0.93 (0.002) | 0.91 (0.002) |
| SVM | Central |  | 0.96 (0.003) | 0.92 (0.005) | 0.89 (0.006) | 0.78 (0.008) |
|  | Federated | IID | 0.96 (0.003) | 0.92 (0.005) | 0.89 (0.006) | 0.78 (0.008) |
|  |  | CI | 0.96 (0.003) | 0.91 (0.005) | 0.89 (0.005) | 0.78 (0.008) |
|  |  | SI | 0.96 (0.003) | 0.92 (0.005) | 0.89 (0.005) | 0.78 (0.008) |
| FNN | Central |  | 0.95 (0.000) | 0.90 (0.004) | 0.90 (0.013) | 0.82 (0.006) |
|  | Federated | IID | 0.95 (0.005) | 0.90 (0.005) | 0.91 (0.008) | 0.84 (0.009) |
|  |  | CI | 0.95 (0.001) | 0.90 (0.005) | 0.91 (0.008) | 0.84 (0.009) |
|  |  | SI | 0.93 (0.010) | 0.90 (0.005) | 0.91 (0.008) | 0.84 (0.009) |
| CNN | Central |  | 0.94 (0.027) | 0.82 (0.041) | 0.90 (0.013) | 0.75 (0.05) |
|  | Federated | IID | 0.93 (0.011) | 0.83 (0.030) | 0.92 (0.008) | 0.83 (0.03) |
|  |  | CI | 0.93 (0.013) | 0.83 (0.002) | 0.92 (0.008) | 0.83 (0.03) |
|  |  | SI | 0.93 (0.010) | 0.83 (0.030) | 0.92 (0.008) | 0.83 (0.03) |
| GBDT | Central |  | 0.98 (0.000) | 0.97 (0.000) | 0.96 (0.0) | 0.94 (0.000) |
|  | Federated | IID | 0.97 (0.002) | 0.96 (0.001) | 0.95 (0.002) | 0.93 (0.001) |
|  |  | CI | 0.97 (0.002) | 0.94 (0.001) | 0.95 (0.001) | 0.92 (0.001) |
|  |  | SI | 0.97 (0.002) | 0.96 (0.001) | 0.95 (0.001) | 0.93 (0.001) |
