# Supplementary material for: A comprehensive experimental comparison between federated and centralized learning
Source: Database (Oxford). 2025 Mar 19;2025:baaf016. doi: 10.1093/database/baaf016 (PMC11928227; doi:10.1093/database/baaf016)
Supplement: baaf016_Supp [file baaf016_supp.zip › Supplemental Appendix.docx]

# Appendix

## Relation between clients and learning rate (for linear models)

The local model update from fedAVG (as used during this paper) is given by SGD

*W_t_^i^*
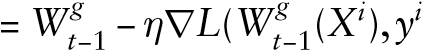
). (A.1)

These local model updates are then combined into a new global model as follows:

*N*

*Wtg* = 1 ∑*siWti*, (A.2)

*S i*=1

where *N* is the amount of clients, *s^i^* the dataset size of client *i*, and *S*
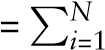
*s^i^*. If we combine equations A.1 and A.2, we get

*N*

*W_t_^g^* = ^1^*S* ∑*i*=1 {*s^i^*[*W_t_^g^*_−1_ −𝜂∇*L*
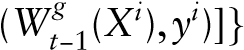
. (A.3)

By doing some refactoring, equation A.3 becomes

*N n*

*Wtg* =1
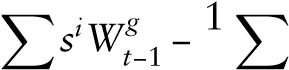
 *s*
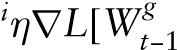
(*Xi*,*yi*)]

*S i*=1 *S i*=0

*g n n*


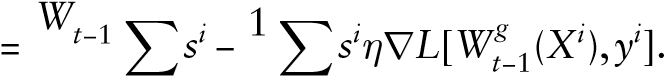
 *S i*=1 *S i*=1

Since *S* = ∑*^n^_i_*_=1_ *s^i^*, we get

*N*

*Wtg* = *Wtg*−1 − 1*S* ∑*i*=0 *si*𝜂∇*L*
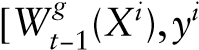
]. (A.6)

The loss function can be written as a sum of the loss over all samples in the local dataset, i.e.

*si*

*L*(*W_t_^g^*(*X^i^*),*y^i^*) = ∑*L*(*W_t_^g^*(*x^i^_j_*),*y^i^_j_*), (A.7)

*j*=0

with *x^i^_j_* being the *j^th^* sample in dataset *X^i^* and *y^i^_j_* its corresponding label. Combining equation A.6 and A.7, we get

*N s^i^*

*Wtg* =*Wtg*−1 − 1*S* ∑*i*=0 *si*𝜂∇∑*j*=0 *L*(*Wtg*(*xji*)*yij*) (A.8)

*N s^i^*


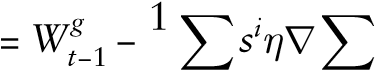
*S i*=0 *j*=0 *Lij*,

where *L^i^_j_* is used as shorthand for *L*(*W_t_^g^*(*x^i^_j_*),*y^i^_j_*). From here we can work towards showing the relation between the amount of clients and learning rate. There are two separate assumptions that can be made, which lead to similar analyses with the same result. Both analyses will be given. The options are as follows:

1. Assume that each client holds an approximately equal amount of samples, i.e. *s*^1^ = *s*^2^ = ... = *s^i^* for each *i* ∈ *N*
2. Assume that each client gives an equivalent model update each round, i.e.
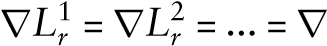
*L^i^_r_* for each *i* ∈ *N*

Note that the second assumption is easiest to satisfy if the samples are IID distributed. However, although unlikely, a scenario is possible in which one clients holds only a few samples which result in a large loss each, whereas most clients hold many more samples with only a small loss per sample. Therefore, it is not strictly necessary to assume IID distribution with assumption 2.

## Using assumption 1

With assumption 1, we can rewrite *s^i^* as

*s*^1^ = *s*^2^ = ... = *s^i^* = *^S^* . (A.10) *N*

Now, using equation A.10 with equation A.8, we get

*N s^i^*

*Wtg* =*Wtg*−1 − 1*S* ∑*i*=0 *NS* 𝜂∇∑*j*=0 *L*(*Wtg*(*xji*)*yji*) (A.11)

*N s^i^*

= *Wtg*−1 − *N*𝜂∇∑*i*=0 ∑*j*=0 *L*(*Wtg*(*xij*),*yij*).

In order to compare with a centralized case, let us assume a setting in which one single client holds all *S* datapoints. A similar (no batch learning) SGD step of such a client can be described as

| *W_t_^c^* = *W_t_*_−1_ −𝜂∇*L*(*W_t_*_−1_(*X*),*y*),  where *W_t_^c^* is the central model at epoch *t* and | (A.13) |
| --- | --- |
| *N X* = ∑*X^i^* | (A.14) |

*i*=1

**Algorithm** **A3.** Federated PCA

1: **input: glob** (bool), *n*_pca_ (int)

2: **On all clients** *i***:** *X*_norm_*^i^* = normalize(*X^i^*, glob) ⊳ *normalize either using global or local mean, see Supplementary Algorithm 4*

3: **On all clients** *i***:** *U^i^*,∑*^i^*,*V^i^* = SV(*X*_norm_*^i^* )

4: **On all clients** *i***:** send *U^i^*, ∑*^i^* to server

5: **On server:** *US^g^* = concat(*U*^1^ *∑^1^,...,*U^N^* *∑*^N^*)

6: **On server:** *U^g^*,∑*^g^*,*V^g^* =SVD(*US^g^*) *g*

7: **On server:** send *U*_0:_*_n_pca* to all clients

8: **On all clients** *i***:** *X_pca_^i^* = *X^i^* **U*_0:_*^g^ _npca_*

**Algorithm** **A4.** Normalization options for fedPCA

1: **input: glob**(bool)

2: **if** glob == True **then**

3: **On Server:** request metadata from all clients *i*

4: **On all Clients:** *mean^i^* = *mean*(*X^i^*),*s^i^* = *length*(*X^i^*) ⊳ *Send back local mean and dataset size*

5: **On server:** Collect *mean^i^*, *std^i^* and *s^i^* for all i

6: **On server:** *S*
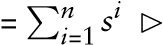
 *Calculate total sample size*

7: **On server:** *mean^g^* = ^1^*S* ∑*^n^_i_*_=1_ *s^i^* **mean^i^* ⊳ *Calculate global mean*

8: **On server:** send *mean^g^* to all clients,

9: **On all Clients:** 𝜎*^i^*
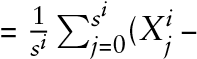
*mean^g^*)^2^ ⊳ *calculate partial variance on each client*

10: **On all Clients:** send 𝜎*^i^* to server

11: **On server:** 𝜎*^g^*
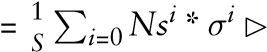
 *calculate global variance*

12: **On server:** send 𝜎*^g^* to all clients

13: **On all Clients** *i***:** *X_norm_^i^* = *Xi*−√*mean*_𝜎_*g g*

14: **else**

15: **On all clients** *i* **do:** *mean^i^* = *mean*(*X^i^*), 𝜎*^i^* = *var*(*X^i^*) ⊳ *calculate local mean and variance*

*^i^* = *X^i^*−*mean^i^*

16:

*norm*

√

𝜎

*i*

17:

**end if**

**On all clients** *i* **do:** *X*

is the concatenation of all local datasets *X^i^*. Combining A.13 and A.14, we get

*N*

*W_t_c*
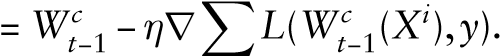


*i*=1

Now using equation A.7, we can get

*N s^i^*

*Wtc* = *Wtc*−1 −𝜂∇∑∑*L*(*Wtc*−1(*xij*),*y*). (A.16)

*i*=1 *j*=1

Comparing equations A.11 and A.16, it becomes clear that they are equivalent, except for the factor ^1^ in equation A.11,

*N*

which effectively lowers the learning rate of the federated case by a factor *N* as compared to its central counterpart.

## Using assumption 2

For this analysis, it is convenient to use equation A.6 as a starting point instead of equation A.8:

*N*

∑*s*

*Wtg* = *Wtg*−1 − 1*_S i_*_=0_ *i*𝜂∇*Li*,

where we use *L^i^* as shorthand for *L*
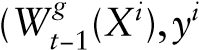
). Since we assume that ∇*L*^1^ = ∇*L*^2^ = ∇*L^i^* for all *i* ∈ *N*, we can remove the dependency of *i*, i.e.

*N N*

*Wtg* = *Wtg*−1 −
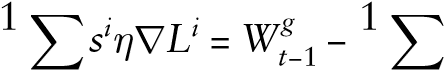
*S i*=0 *S i*=0 *si*𝜂∇*L*.

Since *S* = ∑*^N^_i_*_=0_ *s^i^*, we now get

*N*

*Wtg* = *Wtg*−1 − ∑*N*1 *si* ∑*si*𝜂∇*L* = *Wtg*−1 −𝜂∇*L*.

*i*=0 *i*=0

Compared to the central case, we start off with equation A.16:

*N s^i^ N*

*Wtc* = *Wtc*−1 −𝜂∇∑∑*L*(*Wtc*−1(*xji*),*y*) = *Wtc*−1 −𝜂∇∑*Li*,

*i*=1 *j*=1 *i*=1

where we once again use *L^i^*
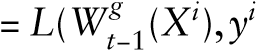
). Once again, using *L^i^* = *L*, we arrive at

*N N*

*Wtc* = *Wtc*−1 −𝜂∇∑*Li* = *Wtc*−1 −𝜂∇∑*L*
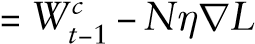
.

*i*=1 *i*=1

## SCAFFOLD

The SCAFFOLD algorithm was created as a means to increase the performance of the earlier discussed fedAVG, specifically with respect to a non-IID setting. A non-IID setting means that the distribution of classes between different clients is not similar, i.e. some clients only have access to a very low amount (in some cases even zero) of samples of certain classes. The problem that arises in fedAVG within the non-IID setting can be intuitively described as a drift, meaning that the global parameters do not or slowly converge to the optimum. In order to combat this drift, (12) introduces a new algorithm for Stochastic Controlled Averaging, called SCAFFOLD.

The main addition in SCAFFOLD is the introduction of a control variate for all clients and for the server. This control variate consists of a set of values with the same structure as the set of parameters (i.e. it has one value per parameter). Intuitively, it denotes the direction (and magnitude) of the local update of the said parameter. If this direction is different from many other clients, the control variate is used to compensate for that difference, which decreases the aforementioned drift.

The general setup of SCAFFOLD is similar to that of fedAVG. The algorithm consists of rounds, which consists of a global part at the server, and a local part which happens at all clients simultaneously. At the beginning of each round, the server sends the global model to all clients, as well as its own control variate c. Now, each client makes a pass over its local data to calculate the gradient over its loss function. This also happens in fedAVG, as part of the SGD step. After the gradient has been determined, the parameters get updated as follows:

*Wri*+1 = *Wrg* −𝜂∇*L*(*X*train*i* ,*yi*train)+*crg* −*cri*. (A.22)

*c*_0_*^g^* and *c_r_^i^* have been initialized as all zero, as described in (12). Note that if both *c^g^* and *c^i^* are all zero, equation A.22 becomes standard SGD, and SCAFFOLD becomes fedAVG for the local step. After the model has been updated, each client also needs to update its control variate. This is done according to the following equation:

*cri*+1 = *cri* −*crg* + 𝜂1*l Wri* −*Wri*+1.

Finally, *W_r_^i^*_+1_ is sent back to the server. Once the server has received *W_r_^i^*_+1_ for all *i* ∈ *N*, it aggregates the local updates into a new iteration of the global model. This update is executed as follows:

*Wrg*+1 = *Wrg* + 𝜂*Ng* *∑*iN*=1 *Wi* −*Wri*, *r*+1

which is equivalent to the global update of federated averaging, if 𝜂*_g_* is set to 1 (which is the case during all experiments described in this report). Different from fedAVG is the need to update *c^g^* as well, which is done according to the following equation:

*N*

*crg*+1 = *crg* + *N*1 *∑*i*=1
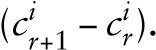


Once this is done, the next round starts by the server sending out the updated model parameters and *c*. A big difference with fedAVG is that SCAFFOLD is a ‘stateful’ algorithm, with the control variates functioning as some sort of state for clients and server.

## Federated PCA

After some initial testing, it turned out that the high dimensionality of the AML datasets was problematic for model convergence. To reduce the dimensionality, a PCA was applied, and the first 100 principal components were chosen to represent the samples in a federated way, described in Supplementary Algorithm 3. Our implementation is a specific case of the incremental federated PCA as proposed in (25) (pseudocode 3), with the increment size being the dataset size (resulting in an approach that is not incremental), as this was fast enough for our experiments. Two versions of normalizing were explored, using a global or a local mean and variance (pseudocode 4). The local normalization was deemed to perform a lot better, as it also serves as a *z*1-normalization.
