# Supplementary figures and images for: A comprehensive experimental comparison between federated and centralized learning
Source: Database (Oxford). 2025 Mar 19;2025:baaf016. doi: 10.1093/database/baaf016 (PMC11928227; doi:10.1093/database/baaf016)

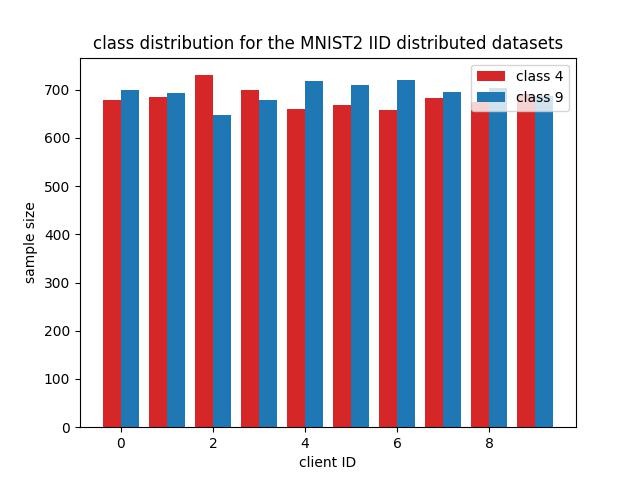

Supplement: baaf016_Supp [file baaf016_supp.zip › suppl_data/2c_IID_dist.jpg]

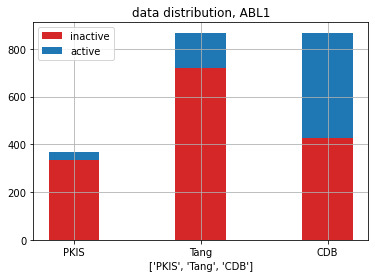

Supplement: baaf016_Supp [file baaf016_supp.zip › suppl_data/ABL1_dist.jpg]

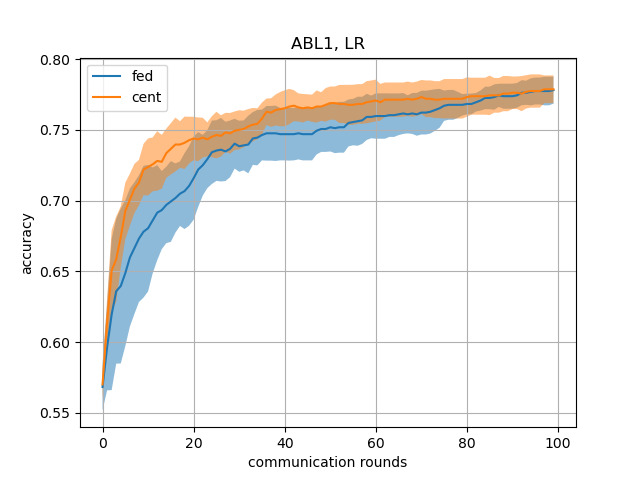

Supplement: baaf016_Supp [file baaf016_supp.zip › suppl_data/ABL1_LR.jpg]

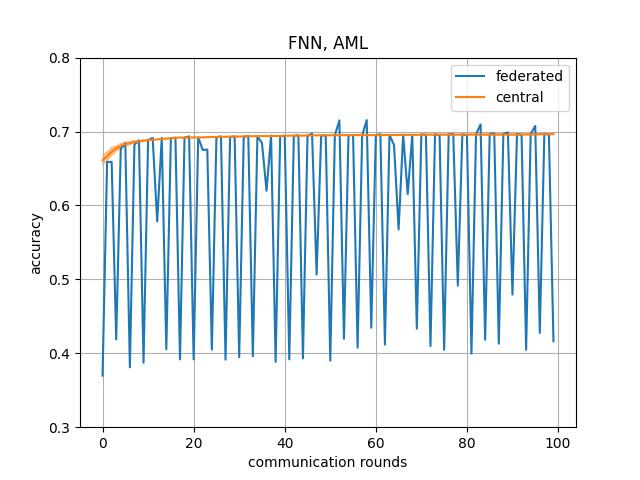

Supplement: baaf016_Supp [file baaf016_supp.zip › suppl_data/AML_before.jpg]

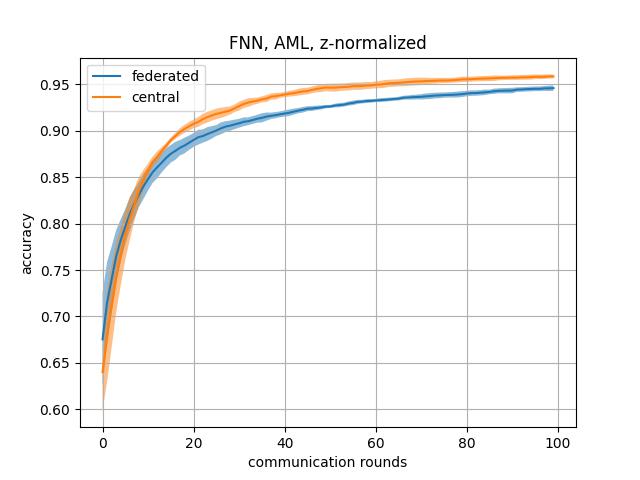

Supplement: baaf016_Supp [file baaf016_supp.zip › suppl_data/AML_revisited.jpg]

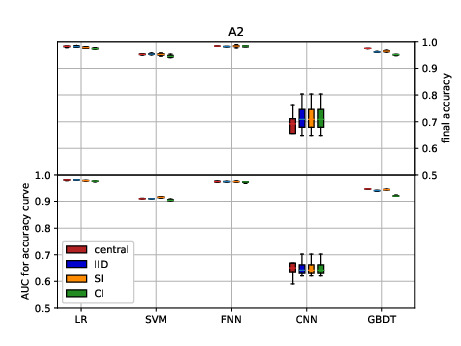

Supplement: baaf016_Supp [file baaf016_supp.zip › suppl_data/AUC_A2.jpg]

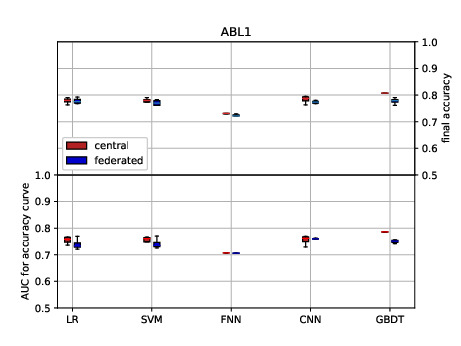

Supplement: baaf016_Supp [file baaf016_supp.zip › suppl_data/AUC_ABL1.jpg]

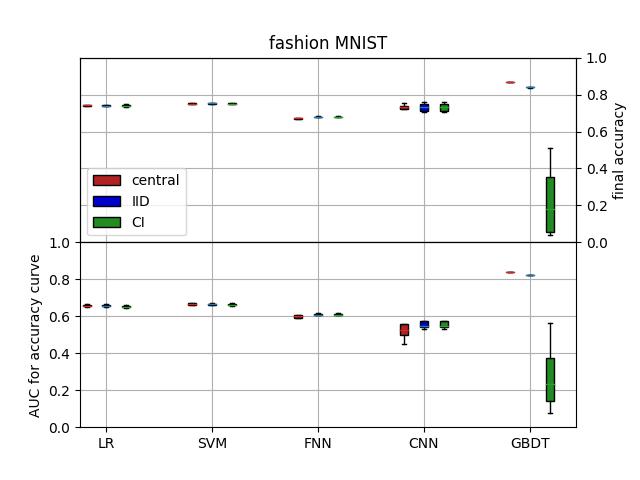

Supplement: baaf016_Supp [file baaf016_supp.zip › suppl_data/AUC_fashionMNIST.jpg]

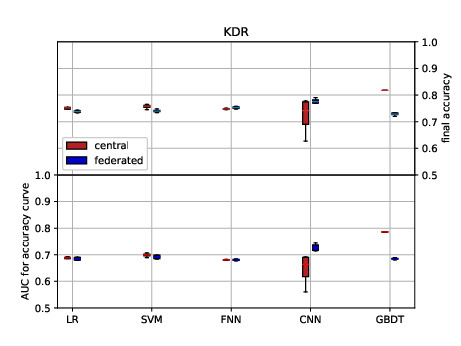

Supplement: baaf016_Supp [file baaf016_supp.zip › suppl_data/AUC_KDR.jpg]

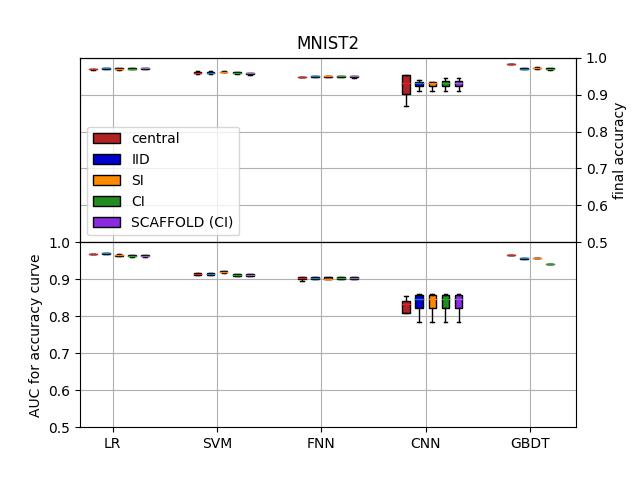

Supplement: baaf016_Supp [file baaf016_supp.zip › suppl_data/AUC_MNIST2.jpg]

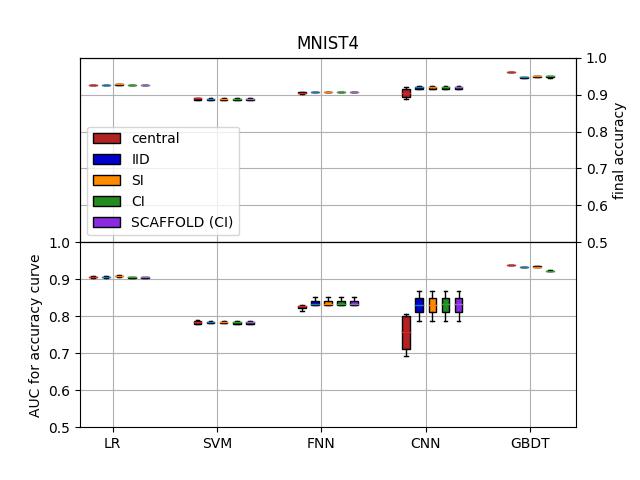

Supplement: baaf016_Supp [file baaf016_supp.zip › suppl_data/AUC_MNIST4.jpg]

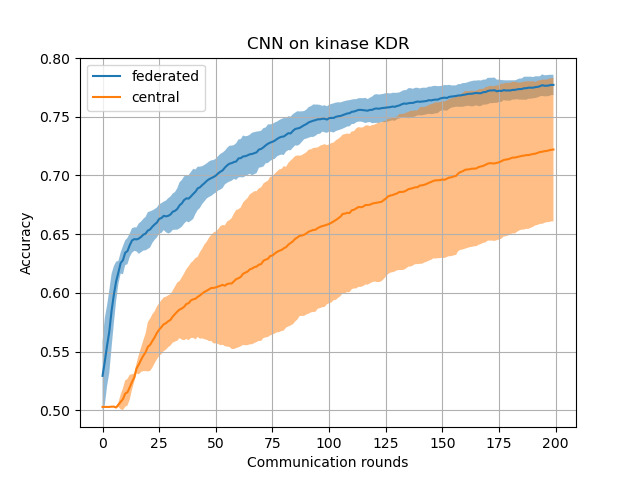

Supplement: baaf016_Supp [file baaf016_supp.zip › suppl_data/CNN_KDR.jpg]

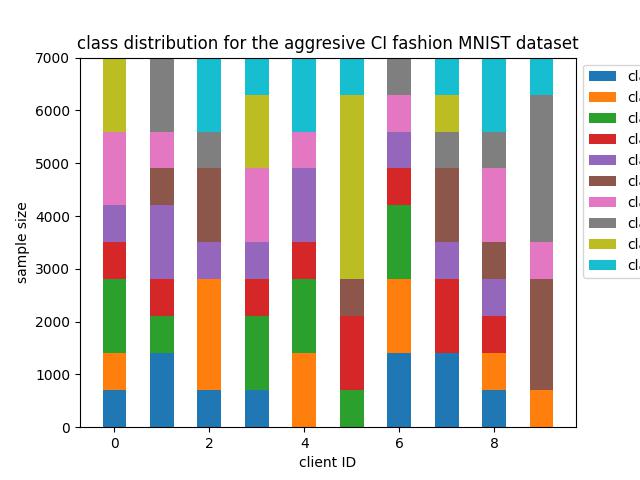

Supplement: baaf016_Supp [file baaf016_supp.zip › suppl_data/fashion_MNIST_ci_dist.jpg]

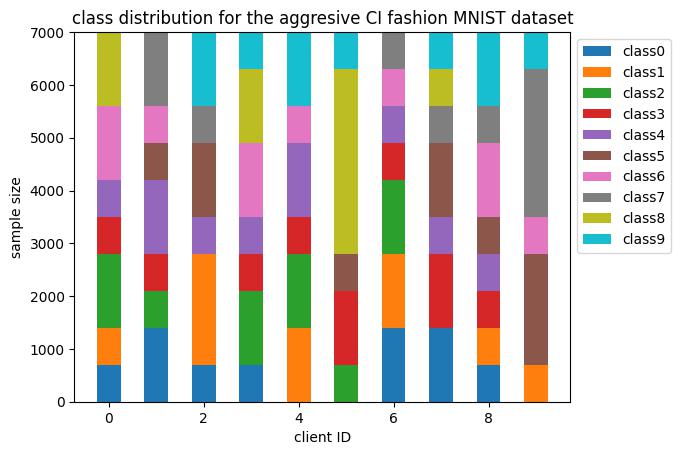

Supplement: baaf016_Supp [file baaf016_supp.zip › suppl_data/fashion_MNIST_ci_dist_stacked.jpg]

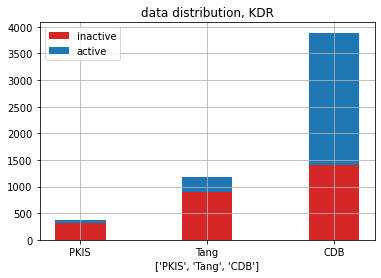

Supplement: baaf016_Supp [file baaf016_supp.zip › suppl_data/KDR_dist.jpg]

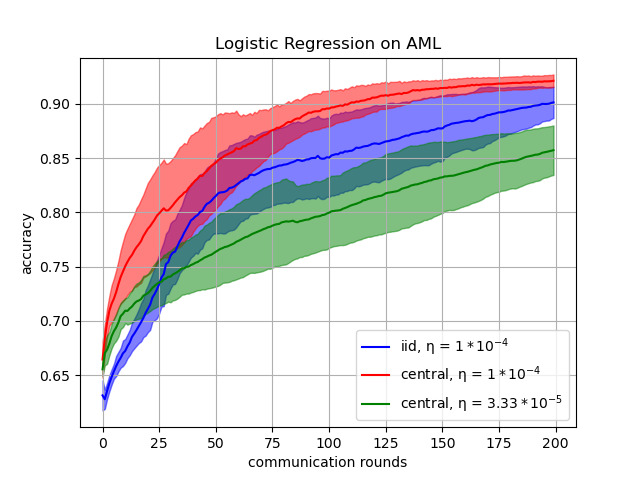

Supplement: baaf016_Supp [file baaf016_supp.zip › suppl_data/LR_AML.jpg]

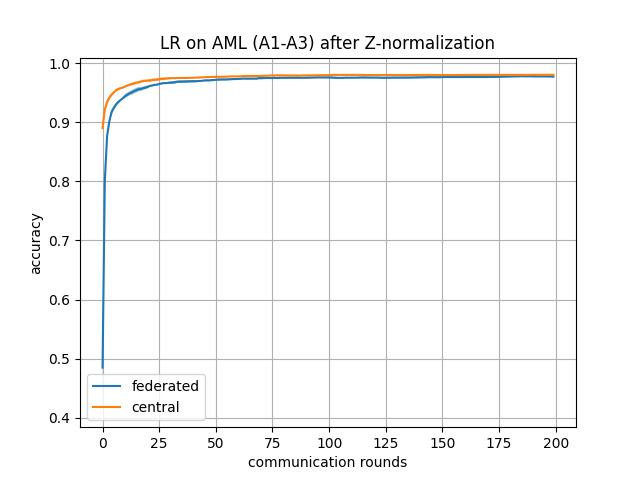

Supplement: baaf016_Supp [file baaf016_supp.zip › suppl_data/LR_AML_post.jpg]

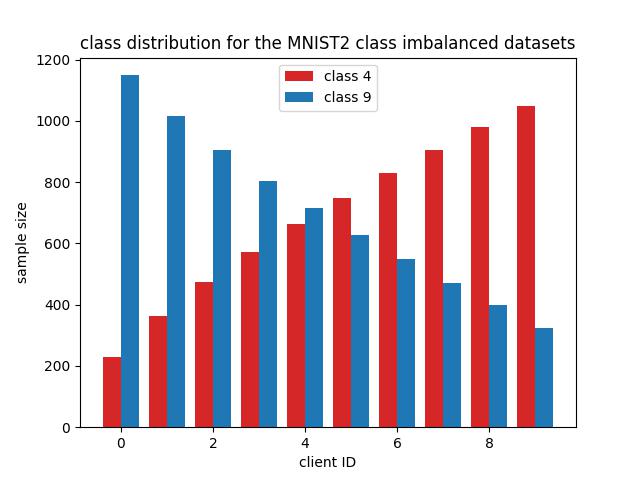

Supplement: baaf016_Supp [file baaf016_supp.zip › suppl_data/MNIST2_ci_dist.jpg]

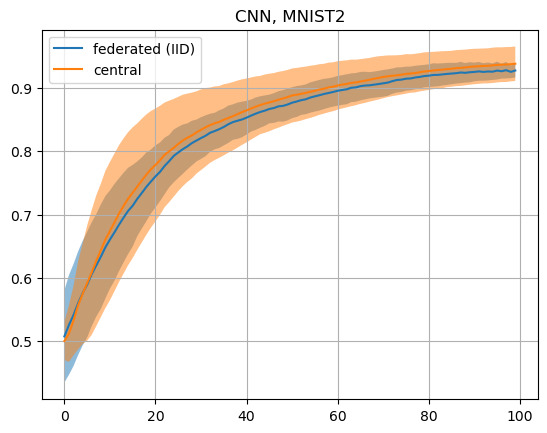

Supplement: baaf016_Supp [file baaf016_supp.zip › suppl_data/MNIST2_CNN.jpg]

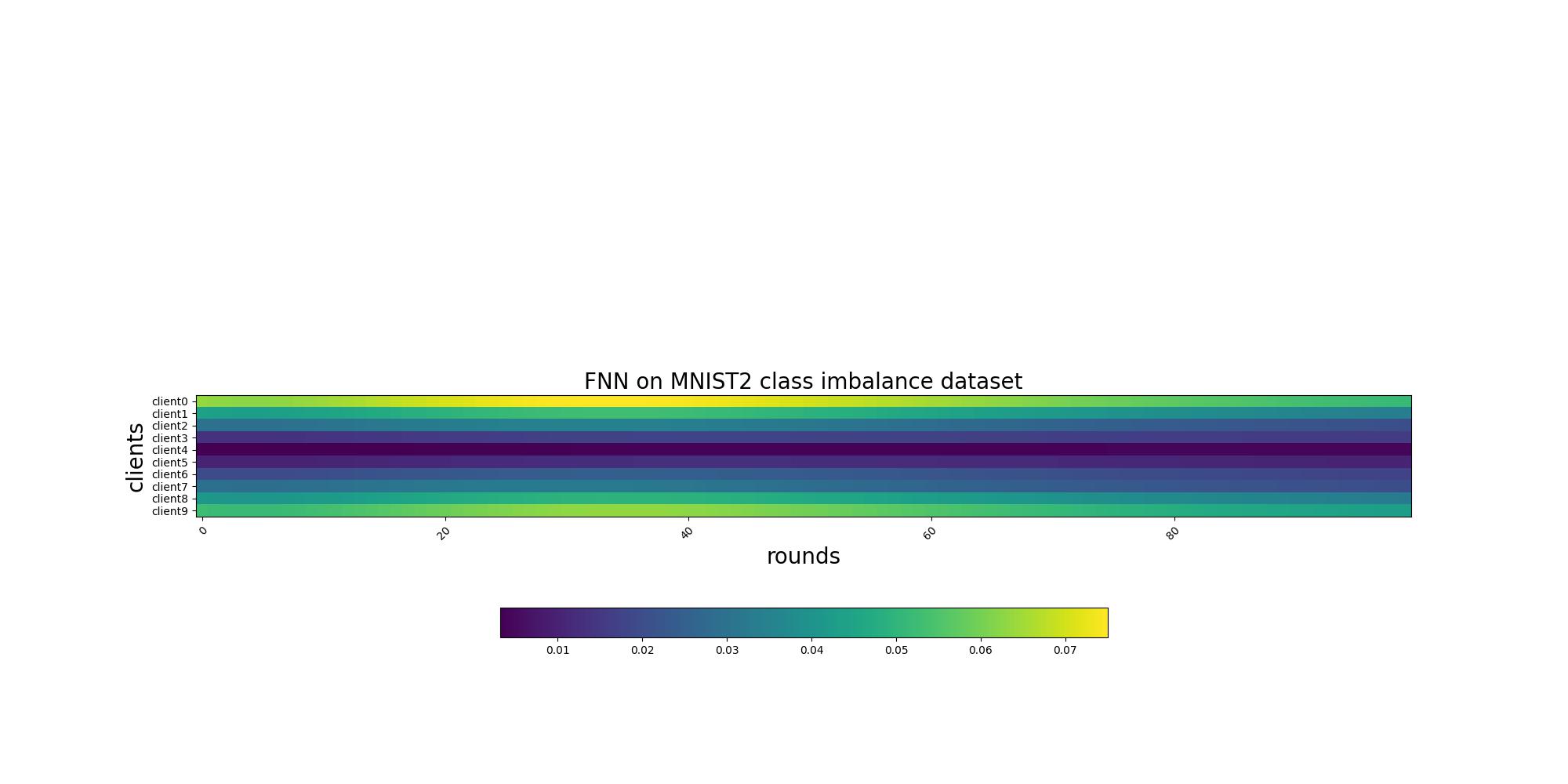

Supplement: baaf016_Supp [file baaf016_supp.zip › suppl_data/MNIST2_FNN_CI_heatmap.jpg]

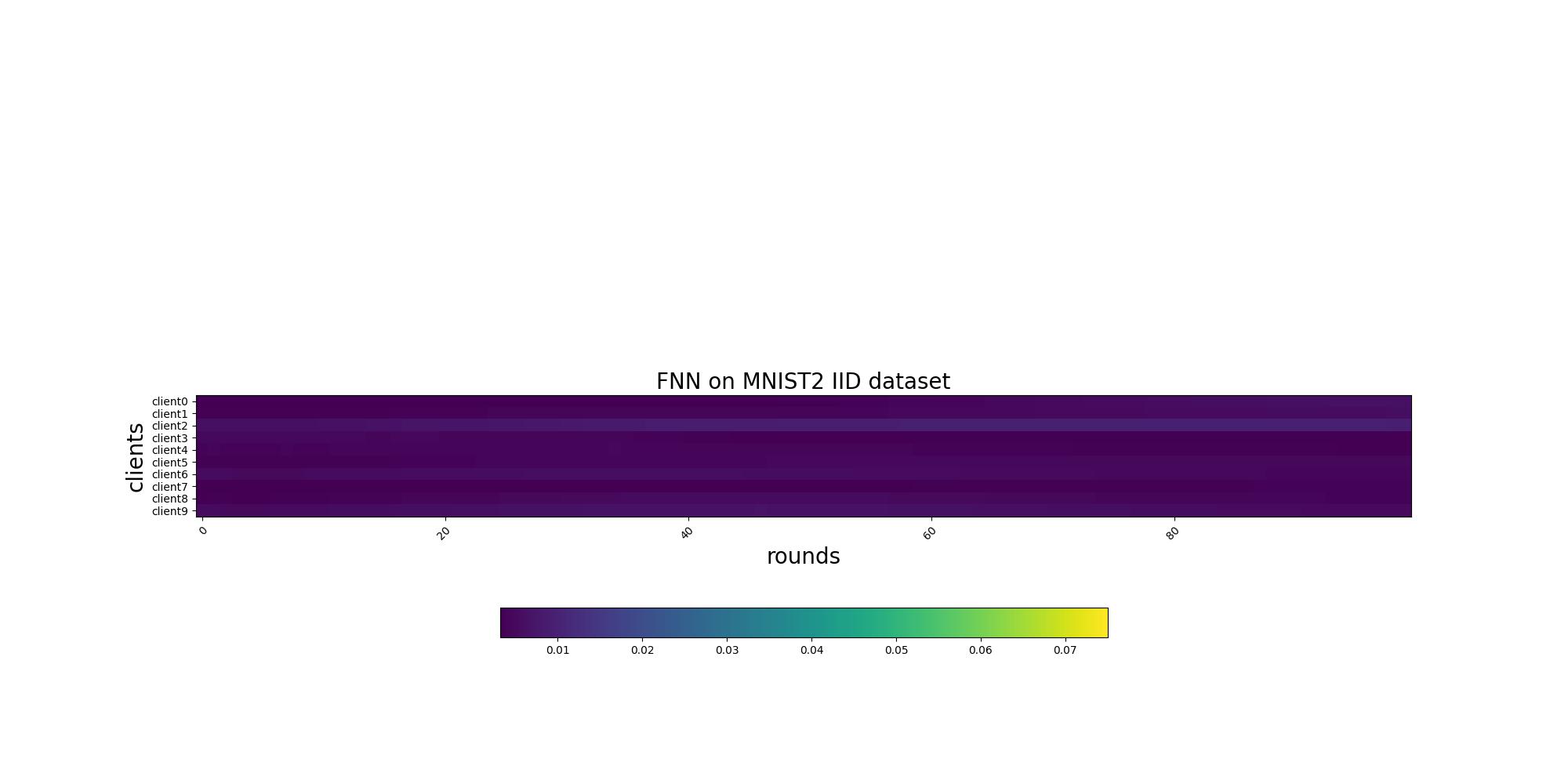

Supplement: baaf016_Supp [file baaf016_supp.zip › suppl_data/MNIST2_FNN_IID_heatmap.jpg]

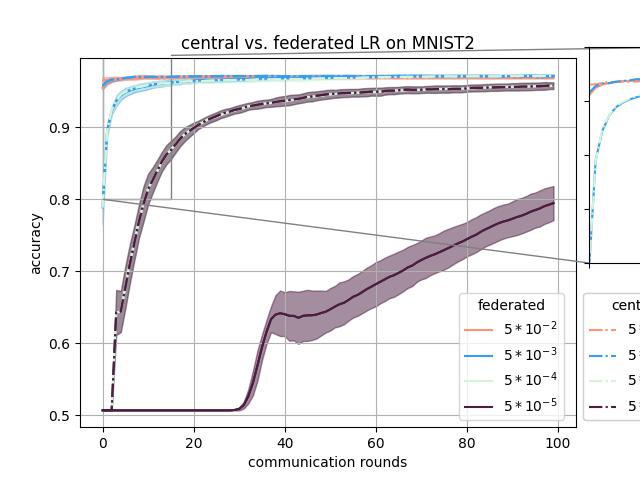

Supplement: baaf016_Supp [file baaf016_supp.zip › suppl_data/MNIST2_lrate.jpg]

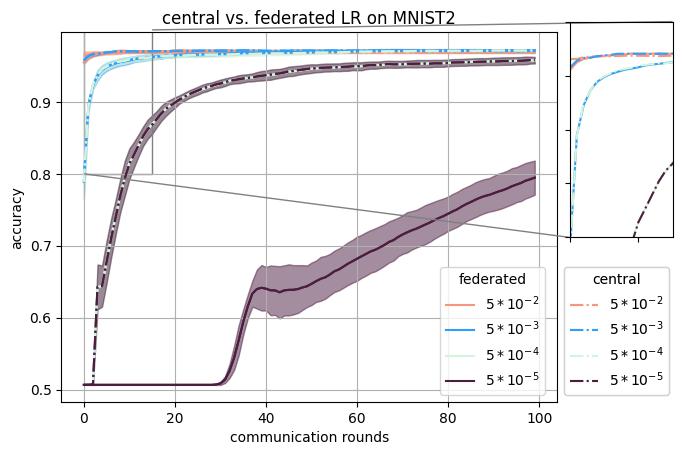

Supplement: baaf016_Supp [file baaf016_supp.zip › suppl_data/MNIST2_lrate2.jpg]

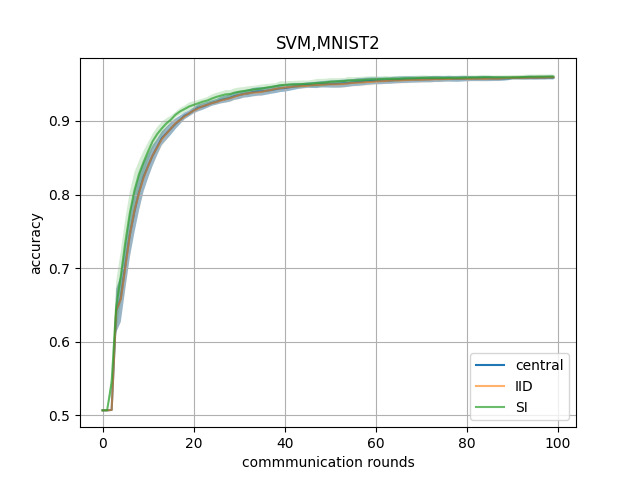

Supplement: baaf016_Supp [file baaf016_supp.zip › suppl_data/MNIST2_SVM.jpg]

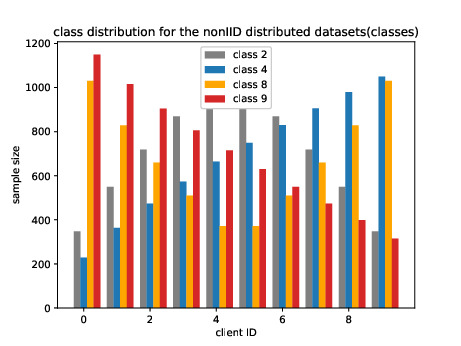

Supplement: baaf016_Supp [file baaf016_supp.zip › suppl_data/MNIST4_ci_dist.jpg]

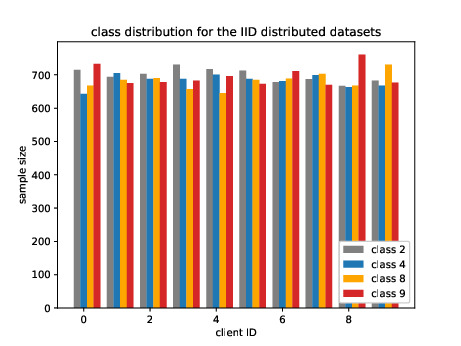

Supplement: baaf016_Supp [file baaf016_supp.zip › suppl_data/MNIST4_IID_dist.jpg]

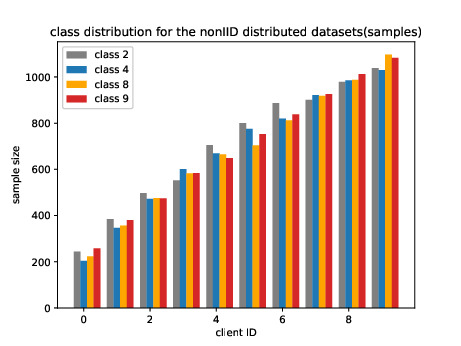

Supplement: baaf016_Supp [file baaf016_supp.zip › suppl_data/MNIST4_SI_dist.jpg]

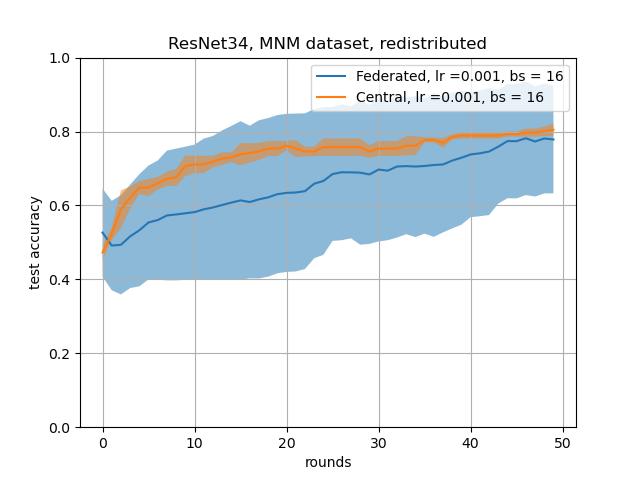

Supplement: baaf016_Supp [file baaf016_supp.zip › suppl_data/MNM.jpg]

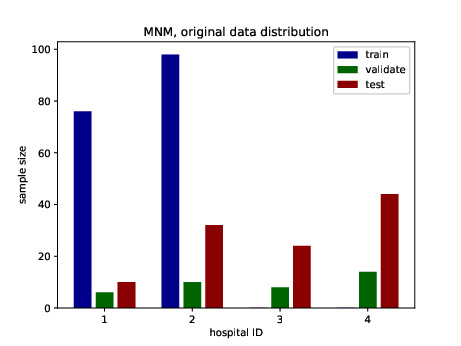

Supplement: baaf016_Supp [file baaf016_supp.zip › suppl_data/MNM_orig_dist.jpg]

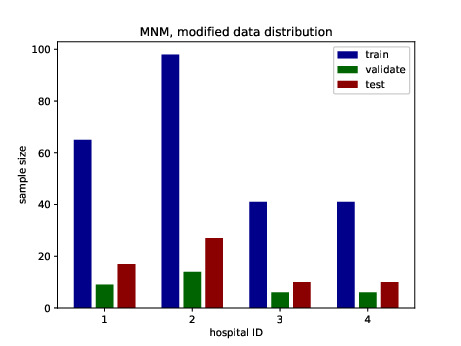

Supplement: baaf016_Supp [file baaf016_supp.zip › suppl_data/MNM_strat_dist.jpg]

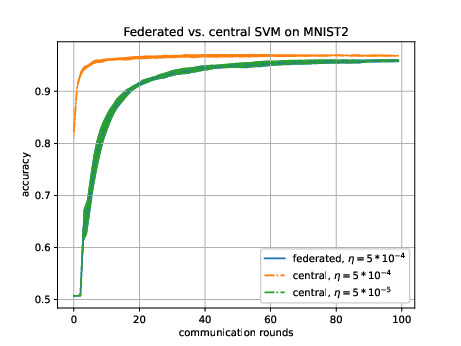

Supplement: baaf016_Supp [file baaf016_supp.zip › suppl_data/SVM_lrates.jpg]

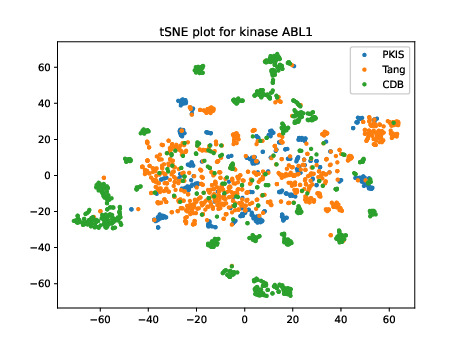

Supplement: baaf016_Supp [file baaf016_supp.zip › suppl_data/tsne_ABL1.jpg]

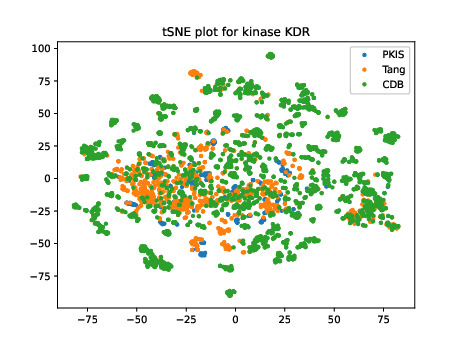

Supplement: baaf016_Supp [file baaf016_supp.zip › suppl_data/tsne_KDR.jpg]
